# Worse inflammatory profile in omnivores than in vegetarians associates with the gut microbiota composition

**DOI:** 10.1186/s13098-017-0261-x

**Published:** 2017-08-15

**Authors:** Ana Carolina Franco-de-Moraes, Bianca de Almeida-Pititto, Gabriel da Rocha Fernandes, Everton Padilha Gomes, Alexandre da Costa Pereira, Sandra Roberta G. Ferreira

**Affiliations:** 1 Department of Epidemiology, School of Public Health, University of Sao Paulo, Av. Dr. Arnaldo, 715, Sao Paulo, SP Zip code 01246-904 Brazil; 2 Department of Preventive Medicine, Federal University of Sao Paulo, Rua Botucatu, 720, Sao Paulo, SP Zip code 04023-900 Brazil; 3 Oswaldo Cruz Foundation, René Rachou Research Center, Av. Augusto de Lima, 1715, Belo Horizonte, MG Zip code 30190-002 Brazil; 4 Laboratory of Genetics and Molecular Cardiology, Heart Institute, Medical School, University of Sao Paulo, Av. Dr. Eneas de Carvalho Aguiar, 44, 10°. andar, Sao Paulo, SP Zip code 05403-000 Brazil

**Keywords:** Gut microbiota, Diet, Biomarkers, Inflammation, Insulin resistance

## Abstract

**Aims:**

To describe the abundance of major phyla and some genera in the gut microbiota of individuals according to dietary habits and examine their associations with inflammatory markers, insulin resistance, and cardiovascular risk profile.

**Methods:**

A total of 268 non-diabetic individuals were stratified into groups of dietary types (strict vegetarians, lacto-ovo-vegetarians, and omnivores). The taxonomic composition and phylogenetic structure of the microbiota were obtained through the analysis of the 16S rRNA gene. Samples were clustered into operational taxonomic units at 97% similarity using GreenGenes 13.5 database. Clinical, biochemical, and circulating inflammatory markers were compared by ANOVA or Kruskal–Wallis test.

**Results:**

The sample (54.2% women, mean age 49.5 years) was composed of 66 strict vegetarians, 102 lacto-ovo-vegetarians and 100 omnivores. Considering the entire sample, the greatest abundant phyla were *Firmicutes* (40.7 ± 15.9%) and *Bacteroidetes* (39.5 ± 19.9%), and no difference in abundances was found between individuals with normal and excess weight. Stratifying by dietary types, the proportion of *Firmicutes* was lower and of *Bacteroidetes* was higher in strict vegetarians when compared to lacto-ovo-vegetarians and omnivores. At the genus level, strict vegetarians had a higher *Prevotella* abundance and *Prevotella/Bacteroides* ratio than the other groups. They also had a lower proportion of *Faecalibacterium* than lacto-ovo-vegetarians, and both vegetarian groups had higher proportions than did omnivores. *Succinivibrio* and *Halomonas* from the *Proteobacteria* phylum were overrepresented in omnivores. The omnivorous group showed higher values of anthropometric data, insulin, HOMA-IR, and a worse lipid profile. Inflammatory markers exhibited a gradual and significant increase from the vegetarians and lacto-ovo-vegetarians to the omnivorous group.

**Conclusions:**

There are differences in gut microbiota composition of individuals with distinct dietary habits, who differ according to their inflammatory and metabolic profiles. Based on the findings relative to bacteria abundances and on their recognized actions in the metabolism, we suggest that exposure to animal foods may favor an intestinal environment which could trigger systemic inflammation and insulin resistance-dependent metabolic disorders.

## Background

The role of dietary habits for risk and for protection against cardiometabolic diseases is largely recognized [1]. While a high-fat low-fiber western diet has been associated with type 2 diabetes mellitus, dyslipidemia and cardiovascular disease, a vegetarian diet with a beneficial cardiometabolic profile and lower rates of cardiovascular events [2, 3]. Moreover, the ability of diet to decrease all-cause and cardiovascular mortality was demonstrated prospectively in vegetarians from the adventist health study-2 [4]. It is known that the body fat accumulation is an underlying mechanism of cardiometabolic disease by triggering a low-grade inflammation and insulin resistance [5, 6]. More recently, there is a growing interest in the participation of gut bacteria mediating the diet-induced cardiometabolic risk [7–9]. Animal studies have contributed to understanding how bacterial communities influence energy extraction, fat deposition, inflammatory status and insulin sensitivity [10–13].

In humans, numerous factors like delivery type, breastfeeding, antibiotic use and dietary habits have been implicated in the interindividual gut microbiota variability and diseases risk [14–18]. In contrast to the initial studies in lean and obese animals [11], metagenomic analyses have indicated that the identification of relative abundance of the two major phyla, *Firmicutes* and *Bacteroidetes*, may not be sufficient to predict body adiposity and obesity-related diseases [19].

Inconsistent results regarding the initially described increased *Firmicutes/Bacteroidetes* ratio (F/B ratio) in human obesity have motivated deepening the knowledge of the phylogenetic structure of microbial communities in population exposed to different environments [20–22]. Studies in some populations have detected associations of dietary patterns with the microbiota composition and certain chronic diseases [21, 23–25]. Underlying pathways of these associations are not completely clarified but it is recognized the ability of a high-fat diet to increase intestinal permeability allowing lipopolysaccharides access in bloodstream [26–28]. A gut microbiome-induced endotoxemia has been associated with increased risk of type 2 diabetes mellitus [7, 13, 29].

Scarce data are available in South America countries characterized by a large variety of foods and dietary habits. The study ADVENTO—Analysis of diet and lifestyle for cardiovascular prevention in 7th-day Adventists (http://www.estudoadvento.org), conducted in a sample of the Brazilian population, has represented a unique opportunity to investigate how vegetarianism and exposure to animal food are associated with the gut microbiota and cardiometabolic risk profile. We hypothesized that dietary-dependent microbial composition influences inflammatory status, insulin resistance, and cardiovascular risk in individuals undergoing distinct dietary habits.

The aims of this study were: to describe the abundance of major phyla and genera in the gut microbiota of non-diabetic Brazilians classified according to dietary types (strict vegetarian, lacto-ovo-vegetarian, and omnivore); and to compare their inflammatory status, insulin resistance index, and cardiovascular risk profile.

## Methods

### Subjects

A convenience sample of first 300 participants from the ADVENTO (http://www.estudoadvento.org), aged 35–65 years old, was invited to join in this cross-sectional analysis. Exclusion criteria were body mass index (BMI) ≥40 kg/m^2^, diabetes mellitus, history of inflammatory bowel diseases or persistent diarrhea, and use of antibiotics or probiotic or prebiotic supplements within the 2 months prior to data collection. A total of 268 subjects satisfied those criteria and were stratified according to dietary type: strict vegetarian, lacto-ovo-vegetarian, and omnivore. Participants were examined at the Investigation Center of the University of Sao Paulo Hospital from March 2013 to October 2014. After overnight fasting, they visited the Center for clinical examination and biological samples collection.

A trained staff collected dietary and clinical data. Dietary types were defined based on the referred food consumption for the last year. Participants were considered strict vegetarians when consuming no animal product (red meat, poultry, fish, eggs, milk, and dairy products <1 time/month); lacto-ovo-vegetarians when consuming dairy products and/or eggs ≥1 time/month, but no fish or meat (red meat, poultry, and fish <1 time/month); and omnivores if they eat animal products (red meat, poultry, fish, eggs, milk, and dairy products) more than once a month [30, 31].

### Clinical data

Height was obtained using a fixed stadiometer and weight with subjects wearing light clothing and no footwear, placed on a digital scale with 200 kg capacity, accurate to the nearest 100 g. BMI was calculated as weight in kilograms divided by height in meters squared; values ≥25.0 kg/m^2^ was considered weight excess. Waist circumference was measured at the midpoint between the bottom of the rib cage and the top of the iliac crest during minimal respiration. The body composition was measured using a bioelectrical impedance analysis (BIA—InBody230; BioSpace, Seoul, Korea). Blood pressure was taken using a validated oscillometric device (Omron HEM 705CPINT, Omron Health Care, Lake Forest, IL, USA) after a 5-min rest in a sitting position. Three measurements were taken at 1-min intervals. The mean of the blood pressure measurements was used in analysis. Hypertension was defined as blood pressure ≥140/90 mmHg (either systolic or diastolic) or use of antihypertensive medication.

### Analytical methods

Blood samples were collected while fasting and during a 2-h standard 75-g oral glucose tolerance test, and were immediately centrifuged for plasma glucose and lipid determinations. Categories of glucose tolerance were defined according to the American Diabetes Association criteria [32]. Aliquots were frozen at −80 °C for further determinations of insulin and inflammatory markers (C-reactive protein—CRP, lipopolysaccharides—LPS, interleukin-10—IL-10, tumor necrosis factor-alpha—TNF-α and E-selectin). Insulin resistance was estimated using homeostasis model assessment (HOMA-IR) [33]. The HOMA-IR has been largely used as an insulin resistance surrogatein clinical and epidemiological studies, based on its strong correlation with the estimates obtained by the euglycaemic clamp [33–35].

The ratio TNF-α/IL-10 was calculated as index of inflammatory response [36].

Plasma glucose was measured by the hexokinase method (ADVIA Chemistry; Siemens, Deerfield, IL, USA). Plasma insulin was determined by enzyme-linked immunosorbent assay (ELISA) (Siemens, Deerfield, IL, USA). Total and high-density lipoprotein cholesterol (HDL-c) and triglycerides were measured by enzymatic colorimetric assay (ADVIA Chemistry; Siemens, Deerfield, IL, USA), while low-density lipoprotein cholesterol (LDL-c) was calculated by the Friedewald equation. High-sensitivity CRP was determined by immunochemistry (Dade Behring; Siemens, IL, USA), and LPS using an ELISA kit (My Bio Source, San Diego, CA, USA). Concentrations IL-10, TNF-α and E-selectin were simultaneously determined by the Multiplex^®^ (R&D Systems, Minnesota, MN, USA).

### Gut microbiota

Fecal samples were maintained under refrigeration (6 °C) within a maximum of 24 h after collection, and then the aliquots were stored at −80 °C until analysis. DNA was extracted using the Maxwell^®^ 16 DNA purification kit and the protocol carried out in the Maxwell^®^ 16 Instrument according to the manufacturer’s instructions (Promega, Madison, WI, USA). Taxonomic composition and phylogenetic structure of a microbial community were obtained through the analysis of the 16S rRNA gene using the Illumina^®^ MiSeq platform and the V4 region. DNA library construction and sequencing were performed following the manufacturer’s instruction (Illumina, San Diego, CA, USA), and the workflow described by Caporaso et al. [37]. Samples were clustered into operational taxonomic units (OTUs) at 97% similarity with Qiime v1.8 using the GreenGenes 13.5 database.

### Statistical analysis

Descriptive data were expressed as means and standard deviations or medians and interquartile range. Since distributions of some variables were skewed, they were log-transformed before analysis to achieve normality; some values in tables were back-transformed to return to the natural scale. Groups of participants classified according to the three dietary types were compared by ANOVA and Bonferroni post hoc test, or by the Kruskal–Wallis test when indicated. Statistical analyses were carried out with IBM SPSS Statistics, version 22.

Participants were also stratified into two groups, omnivores and non-omnivores (strict plus lacto-ovo vegetarians), and the DESeq2 package was used for comparisons [38]. The package provides log2 fold changes attributable to a given variable over the mean of normalized counts of OTUs. The log2 fold changes represent the comparison against the reference level, which is log2 (Omnivores/Non-omnivores). If differences between omnivores and non-omnivores are equal to zero, it indicates that there is no difference between the means of the groups.

## Results

Among 268 participants, 54.2% were women and 41.4% had weight excess. Stratifying according to dietary type, 66 were strict vegetarians, 102 lacto-ovo-vegetarians, and 100 omnivores, and groups did not differ according to male-to-female ratios and age (49.6 ± 8.5, 49.6 ± 8.6, 49.1 ± 8.2 years, p = 0.878, respectively). The frequencies of weight excess [26% (95% CI 15–37) versus 38% (95% CI 29–47) versus 55% (95% CI 45–65), pre-diabetes [21% (95% CI 11–31) versus 29% (95% CI 20–38) and 36% (95% CI 27–45)], and hypertension [18% (95% CI 9–27) versus 26% (95% CI 17–34) and 33% (95% CI 24–43)] were higher in the omnivores than in lacto-ovo-vegetarians and strict vegetarians, respectively.

**Fig. 1:**
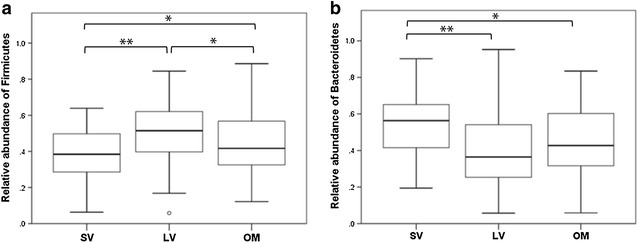
Relative abundance of *Firmicutes* (**a**) and *Bacteroidetes* (**b**) according to dietary types. *Central lines of the box plots* are the means; *box outline* equals 1 SD; *the bar* denotes 2 SD. *SV* strict vegetarian, *LV* lacto-ovo-vegetarian, *OM* omnivore. ANOVA followed by Bonferroni post hoc test. *p < 0.05, **p < 0.005

Thirteen phyla were identified and five were present in all the fecal samples analyzed. Large relative abundances of *Firmicutes* (40.7 ± 15.9%) and *Bacteroidetes* (39.5 ± 19.9%) were found (Fig. 1), followed by *Proteobacteria*. Stratifying by dietary type, the proportion of *Firmicutes* (Fig. 1, panel a) was lower and of *Bacteroidetes* was higher in the strict vegetarian group (Fig. 1, panel b) when compared to lacto-ovo-vegetarians and omnivores. No differences were found in the comparisons of the F/B ratio between subsets of participants with normal and weight excess, considering the whole sample as well as within each dietary type.

**Fig. 2:**
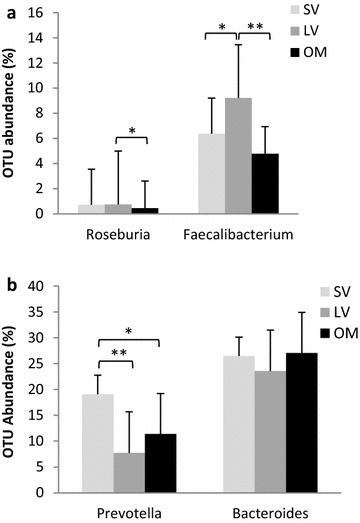
Mean values of abundance (%) of genera belonging to the major phyla. **a** *Firmicutes* phylum: *Roseburia* and *Faecalibacterium* genera. **b** *Bacteroidetes* phylum: *Prevotella* and *Bacteroides* genera. ANOVA followed by Bonferroni post hoc test. *p < 0.05, **p < 0.005

The abundance of OTUs according to dietary types was also compared at genus level. Strict vegetarians had higher rates of *Prevotella* genus that belongs to the *Bacteroidetes* phylum (Fig. 2, panel a), and a higher *Prevotella/Bacteroides* ratio (8.4 ± 0.3) than the other groups. On the other hand, strict vegetarians had a lower proportion of *Faecalibacterium* genus from the *Firmicutes* phylum than lacto-ovo-vegetarians, and both vegetarian groups had higher proportions than in omnivores (Fig. 2, panel b).

**Fig. 3:**
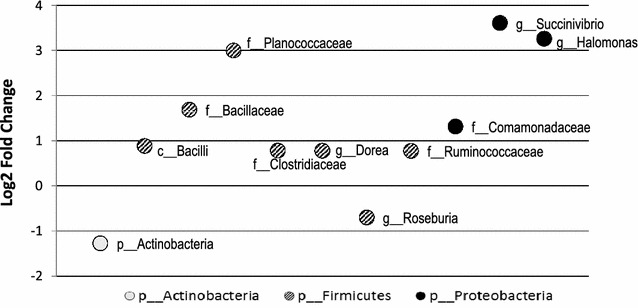
Log2 fold changes in mean reads of selected OTUs in omnivore and non-omnivorous group (strict plus lacto-ovo-vegetarians) at the deepest level identified. DESeq2 detected significant differences between dietary types at p < 0.005. Bacteria represented in *circles at different colors* belong to different phyla

Differences of selected OTUs between omnivores and non-omnivores (strict plus lacto-ovo-vegetarians), members of phyla *Firmicutes*, *Proteobacteria,* and *Actinobacteria,* assessed by the DESeq2, were shown in Fig. 3. The omnivore group showed much higher abundances of genera *Succinivibrio* and *Halomonas*, which belong to *Proteobacteria*, while non-omnivores had more OTUs from *Actinobacteria* and *Roseburia* genus from *Firmicutes*, compared to the counterparts.

The omnivorous group showed higher mean values of anthropometric data and worse lipid profile than the others (Table 1). Non-significant increases in fasting plasma glucose was verified across the groups, but omnivores exhibited higher insulin and HOMA-IR values (p < 0.001). Medians of CRP, LPS, and TNF-α/IL-10 ratio increased gradually from the vegetarian toward the omnivorous group.

**Table 1:** Mean (standard deviation) or medians (interquartile range) of clinical data of participants according to dietary type

	Strict vegetarian	Lacto-ovo-vegetarian	Omnivore	p value
	N = 66	N = 102	N = 100	
Body mass index (kg/m^2^)	23.2 (4.1)	24.4 (3.9)	26.4 (4.7)^b, c^	<0.001
Waist circumference (cm)	79.7 (11.0)	81.7 (10.7)	86.5 (12.9)^b, c^	0.001
Fat mass (%)	27.1 (9.2)	30.2 (8.2)	32.6 (9.1)^b^	0.001
Systolic BP (mmHg)	115 (14)	118 (16)	119 (14)	0.328
Diastolic BP (mmHg)	72 (9)	73 (10)	74 (10)	0.501
Plasma glucose (mg/dL)	91.8 (7.9)	92.3 (7.4)	94.6 (10.1)	0.076
Fasting insulin^a^ (μUI/mL)	6.5 (1.8)	7.4 (1.7)	9.2 (1.7)^b, c^	<0.001
HOMA-IR^a^	1.5 (1.8)	1.7 (1.7)	2.1 (1.8)^b, c^	<0.001
Total cholesterol (mg/dL)	173.9 (36.9)	173.0 (35.6)	185.3 (33.6)^c^	0.028
LDL-cholesterol (mg/dL)	99.3 (31.0)	110.7 (27.2)	113.6 (29.8)^b, c^	0.005
HDL-cholesterol^a^ (mg/dL)	51.7 (1.3)	49.7 (1.3)	50.4 (1.3)	0.614
Triglycerides^a^ (mg/dL)	96.5 (1.55)	92.2 (1.58)	93.4 (1.68)	0.831
C-reactive protein (mg/L)	0.5 (0.4–1.3)	0.8 (0.4–1.7)	1.1 (0.6–2.2)	0.007
Lipopolysaccharides (ng/mL)	31.8 (24.8–45.9)	33.5 (16.2–51.2)	39.5 (23.5–54.7)	0.008
Interleukin-10 (pg/mL)	0.3 (0.2–0.5)	0.3 (0.05–0.5)	0.3 (0.05–0.5)	0.402
TNF-α (pg/mL)	2.7 (1.7–3.6)	2.9 (1.5–5.0)	2.9 (1.9–4.5)	0.423
TNF-α/IL-10	7.3 (4.5–13.1)	10.5 (5.6–18.4)	11.7 (6.5–27.2)	0.015
E-selectin (pg/mL)	28.5 (17.5–53.5)	34.1 (21.0–48.4)	39.4 (23.5–54.7)	0.110

*BP* blood pressure, *HOMA-IR* insulin resistance index, *TNF-α* tumor necrosis factor-alpha, *IL-10* interleukin-10

^a^Log-transformed for analysis; values were back-transformed to return to the natural scale. ANOVA followed by Bonferroni post hoc test or Kruskal–Wallis test

^b^Versus strict vegetarian

^c^Versus lacto-ovo-vegetarian

## Discussion

The hypothesis that diet should influence systemic inflammatory status, insulin resistance and cardiovascular risk profile, via gut microbiota composition, is supported in the present study. In a developing country population of South America, the association between dietary habits and abundance of certain bacterial genera was demonstrated. Non-diabetic Brazilians undergoing distinct dietary types showed that vegetarians had a more favorable gut microbiota composition, characterized by less *Firmicutes* and more *Bacteroidetes* than omnivores. Furthermore, among the *Firmicutes* there was a predominance of genera associated with beneficial phenotypes, while among the *Bacteroidetes* an expected higher proportion of *Prevotella*. These findings suggest that exposure to animal foods could favor a pro-inflammatory intestinal environment, favoring endotoxemia, systemic inflammation and insulin resistance that are involved in the deterioration of the cardiometabolic risk profile.

In agreement with other studies [1–4, 30], the Brazilian strict vegetarian Adventists exhibited a low-risk cardiometabolic profile, particularly when compared to the omnivores. Lower frequencies of obesity, hypertension, and pre-diabetes were observed in the strict vegetarians, similar to the results of the adventist health study-2 [3, 4] and also coherent with reports of fewer cardiovascular events in strict vegetarians [30, 39, 40]. Their food preferences—vegetables, fruits, and whole grains—are rich in fibers and micronutrients, which contribute to reduce oxidative stress, an underlying mechanism of these diseases [41]. Fiber-derived short-chain fatty acids (SCFA), mainly butyrate, acetate, and propionate, are facilitated by the presence of certain commensal bacteria that belong to phylum *Firmicutes* [42–44].

Our findings of lower proportion of *Firmicutes* and higher proportion of *Bacteroidetes* in strict vegetarians compared to omnivores may not be attributed to the differences in body adiposity of the participants. In fact, a meta-analysis did not confirm previous assumptions, based on from animal and human studies, that obesity was associated with an increased F/B ratio [19, 45].

Analyzing particularly the *Firmicutes* subpopulations, we found that strict vegetarians had an increased abundance of the two most recognized butyrate-producing bacteria—*Roseburia* and *Faecalibacterium* [43, 46] in comparison with omnivores. Butyrate is a major energy source for colonocytes and promotes the expression of tight junction proteins, enhancing the intestinal barrier function and consequently, it protects against the LPS translocation [43, 47]. Our finding of lower LPS concentration in the strict vegetarian group is coherent with these effects. Also, the anti-inflammatory action of butyrate due to the nuclear factor κB inhibition in colonic cells [43, 46] is supported by our results since lower values of inflammatory markers, CRP and TNF-α/IL-10 ratio, were detected in the same group. However, our study design precluded establishing cause-effect relationship. Therefore, despite belonging to the *Firmicutes* phylum, *Roseburia* and *Faecalibacterium* genera were shown to be associated with a beneficial metabolic profile in our vegetarians, characterized by lower body adiposity and better lipid profile and insulin resistance index, which are along the same line of previous reports [16, 48].

*Bacteroidetes* were relatively more frequent in the microbiota of strict vegetarians than in omnivores, and their subpopulations were mainly composed of the genera *Prevotella* and *Bacteroides*. A higher *Prevotella/Bacteroides* ratio was seen in the strict vegetarians. These findings are consistent with others that reported higher *Prevotella* abundance in individuals with a plant-based diet and predominance of *Bacteroides* in non-vegetarians [15, 16, 49, 50]. Also, investigations on the interaction between long-term dietary patterns and microbiota using genera clusters found an association of *Prevotella* enterotype with fiber-enriched diets, as well as *Bacteroides* enterotype with protein and animal fat [15]. One study is in disagreement, since no significant difference between the gut microbiota composition of vegetarians and omnivores was observed [47].

Our observations of higher LPS, CRP, TNF-α/IL-10 ratio, and HOMA-IR values in the omnivorous group reinforce previous hypothesis that a saturated fat-enriched diet could induce inflammation and insulin resistance [7, 26, 28]. We speculate that exposure to animal foods could have contributed to alter the gut microbiota composition favoring an increase in LPS and generating endotoxemia. LPS are present in outer membrane of gram-negative bacteria and its ability to reduce the expression of tight junction proteins and increase intestinal permeability were demonstrated [29]. Also, there is evidence that microbiota-derived LPS in circulation, by binding to TLR4, trigger inflammation, deteriorate insulin signaling, and cause metabolic disturbances [7, 51, 52]. TLR4-deficient mice were recognized as resistant to the inflammatory activation induced by obesity or free fatty acids and protected from insulin resistance [52]. We suppose that, in the omnivorous participants, animal food consumption could have favored an enrichment of gram-negative bacteria, increased gut permeability and activation of immune response. Their higher levels of some inflammatory markers corroborate for this pathophysiological mechanism, anticipating the alterations in traditional cardiovascular risk factors. Actually, we detected an overrepresentation in some OTUs (increased Succinivibrio and Halomonas abundances) from the *Proteobacteria* phylum, which is known as a major group of gram-negative bacteria that prefer proteins as main energy source [49]. A similar result has already been reported in another study in which *Proteobacteria* were more abundant in European children fed with western diet compared to Africans fed predominantly with vegetarian diet [16].

Also, overrepresentation of class *Bacilli*, belonged to *Firmicutes* phylum, was verified among omnivores, which could be somehow unexpected since these bacteria are not gram-negative. However, our result is concordant with a report of an association of high abundance of *Bacilli* with the western diet [53] and diabetes [54], both conditions associated with low-grade inflammation and insulin resistance. Inflammatory markers and HOMA-IR were, in fact, higher in our omnivorous participants compared to vegetarians.

In lacto-ovo-vegetarians, we observed a higher proportion of *Firmicutes* and among these, of the *Faecalibacterium* genus. This finding was previously reported [50] and it was suggested that dairy products and eggs might be substrates for these bacteria [55]. Since these participants were exposed to this kind of animal foods and an intermediate cardiometabolic risk level, between the strict vegetarians and omnivores, was expected. Despite clinical parameters within the normal ranges, they already exhibited signs of a pro-inflammatory and reduced insulin sensitivity condition.

The main limitation is related to the cross-sectional design impeding the establishment of temporal or causal relationships. Also, the lack of detailed nutrient information inquiry made our study even more speculative. On the other hand, our study has the strength of reporting data on the gut microbiota at a deep level in a considerable number of individuals consuming with distinct dietary patterns. As far as we know, this approach is unique in South America countries. These data were associated with traditional risk factors and emergent cardiometabolic markers, suggesting possible mechanisms by which diet-mediated bacteria could participate in the genesis of prevalent diseases linked by the insulin resistance.

We called attention to the importance of gut microbiota assessment for understanding how diet participates in the pathogenesis of cardiometabolic diseases. Whether deeper taxonomic classification could provide clues in the investigation of the pathophysiological mechanisms of these complex diseases requires further investigation.

In conclusion, our data support that there are differences in gut microbiota composition of individuals consuming distinct types of diet, who differ according to their inflammatory and metabolic profiles. Based on the findings relative to bacteria abundances and on their recognized actions in the metabolism, we suggest that exposure to animal foods may favor an intestinal environment which could trigger systemic inflammation and insulin resistance-dependent metabolic disorders.
